# The Synergy of Chitosan and Azoxystrobin Against *Fusarium graminearum* Is Modulated by Selected ABC Transporters

**DOI:** 10.3390/ijms26010262

**Published:** 2024-12-30

**Authors:** Pawel Poznanski, Abdullah Shalmani, Pascal Poznanski, Waclaw Orczyk

**Affiliations:** 1 Plant Breeding and Acclimatization Institute—National Research Institute, Radzikow, 05-870 Blonie, Poland; 2 Ludwik Rydygier Collegium Medicum in Bydgoszcz, Nicolaus Copernicus University in Torun, 85-094 Bydgoszcz, Poland

**Keywords:** Amistar, copper oxide, dsRNA, fungicides, Fungimat, metconazole, Micosar, synergy score, tebuconazole, *Fusarium* transcriptome

## Abstract

The development of innovative and effective strategies to combat fungal pathogens is critical to sustainable crop protection. Fungicides have been used for over two centuries, with traditional copper- and sulfur-based formulations still in use due to their broad-spectrum, multisite mode of action, which minimizes the risk of pathogen resistance. In contrast, modern systemic fungicides, though potent, often target a single site of action, leading to the accelerated emergence of resistant fungal strains. This study explores synergistic interactions between chitosan (CS) and selected fungicides, focusing on their antifungal activity against *Fusarium graminearum*. Among the fungicides tested, azoxystrobin (Amistar) exhibited the highest 44.88 synergy score when combined with CS (30 kDa, degree of deacetylation ≥ 90), resulting in significantly improved antifungal efficacy. Furthermore, the combination of CS and Amistar with double-stranded RNA (dsRNA) targeting selected ABC transporter genes further amplified antifungal activity by silencing genes critical for fungal tolerance to treatment. This dual synergy highlights the potential of RNA interference (RNAi) as both a functional tool to investigate fungal physiology and an effective antifungal strategy. These findings reveal a promising and environmentally friendly approach to mitigate resistance while improving fungal control. Furthermore, the remarkable synergy between azoxystrobin and CS presents a novel mechanism with significant potential for sustainable agricultural applications, which warrants further investigation to elucidate its molecular basis.

## 1. Introduction

Exploring synergistic interactions can deepen our understanding of the mechanisms behind the antifungal activities of specific compounds and guide the development of innovative and more effective combinations. Chitosan (CS), a copolymer composed of N-acetyl-D-glucosamine (GlcNAc) and deacetylated D-glucosamine (GlcN), is derived through the partial deacetylation and hydrolysis of chitin, the second most abundant natural polysaccharide after cellulose. Thanks to its distinct molecular characteristics, CS exhibits notable biological activities across various organisms. It has strong antifungal properties, effectively inhibiting the growth of pathogenic fungi and reducing mycotoxin levels in plant tissues [1]. Furthermore, CS is recognized for its ability to stimulate plant immune responses and enhance growth [2,3]. These attributes, combined with its biocompatibility and biodegradability, position CS as a promising candidate for applications in sustainable agriculture [4]. Its proven ability to control *Fusarium* growth, minimize mycotoxin production, and strengthen plant immunity highlights its potential as a powerful solution for managing *Fusarium*-related plant diseases. Fungicides have been utilized for over two centuries to protect crops from fungal pathogens. Traditional copper- and sulfur-based formulations, while relatively less potent, remain in use due to their multisite mode of action, which significantly reduces the risk of fungal resistance [5]. For example, the Miedzian fungicide used in this study contains copper oxychloride as its active ingredient. Copper oxychloride interacts with sulfhydryl groups in proteins, leading to their denaturation and restricting fungal growth [6,7]. In contrast, modern systemic fungicides that target a single site of action are more prone to fostering fungal resistance [8], thereby limiting their long-term utility. Examples of such fungicides used in this study include Amistar, Fungimat, and Micosar. Amistar, which contains azoxystrobin, is a broad spectrum fungicide that disrupts mitochondrial electron transport at the Q_0_ center of cytochrome bc_1_ [9]. Fungimat, with tebuconazole as its active ingredient, belongs to the triazole class of fungicides, which inhibit 14α-demethylase activity, impairing ergosterol synthesis and halting fungal growth [10]. Similarly, Micosar, containing metconazole as the active compound, is another broad-spectrum fungicide that inhibits ergosterol biosynthesis and is highly effective against *Fusarium* head blight (FHB) [11].

ATP-binding cassette (ABC) transporter genes have very broad biological functions such as signal transduction, and they are associated with pathogenesis and fungicide resistance in many species of fungi [12,13,14]. Typically, ABC transporter-based resistance is linked to the upregulation of these genes [15,16,17]. In *Fusarium graminearum (F. graminearum)*, deletion of ABC genes has been shown to decrease virulence and increase susceptibility to azole group fungicides [18].

Our previous research explored the antifungal properties of CS, highlighting its potential to reduce fungal pathogenesis and inhibit mycotoxin production. Notably, significant antifungal activity was observed only in a specific batch of CS characterized by low molecular weight (MW) and a high degree of deacetylation (DD) [1]. In the present study, we observed that the most effective CS sample exhibited synergistic interactions when combined with selected fungicides. This article, therefore, aims to investigate these synergistic effects and elucidate the potential mechanisms underlying such interactions. The widespread reliance on modern, single-site fungicides often accelerates the emergence of resistant fungal strains [8]. Combining antifungal agents with different modes of action, such as site-specific fungicides and broad-spectrum CS, offers a promising strategy to minimize the use of plant-protective agrochemicals and mitigate the development of fungicide-resistant pathogens.

## 2. Results

### 2.1. Antifungal Activity of Selected Fungicides

**Figure 1:**
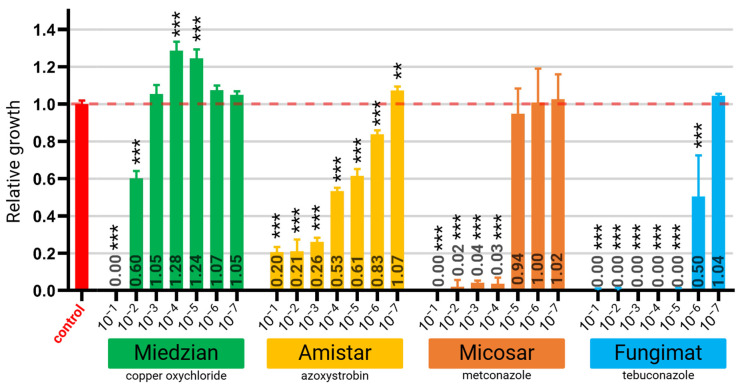
Relative growth of *Fusarium graminearum* in PDB medium supplemented with fungicides: Miedzian Extra 350 SC, Amistar 250 SC, Micosar 60 SL, and Fungimat. Fungal growth was measured as optical density at 450 nm (OD_450_) and expressed as relative values, with the OD_450_ of the control culture in PDB medium normalized to 1.0 (indicated by a red line). Fungicide concentrations are presented as serial dilutions of the original commercial formulations. Statistical significance is indicated as follows: ** *p* ≤ 0.01, *** *p* ≤ 0.001.

The antifungal activities of selected fungicides were evaluated using 96-well microtiter plate culture of *F. graminearum* in potato dextrose broth (PDB) medium. The relative growth of *F. graminearum* in PDB medium supplemented with fungicides was shown as a proportion of its growth in the control PDB medium without fungicides (Figure 1) across series of concentrations (Supplementary Table S3). Miedzian Extra 350 SC containing copper oxychloride as the active ingredient (350 g/L) exhibited the weakest fungicidal or fungistatic activity. Growth inhibition was observed at a 10^−^^1^ dilution of the commercial fungicide, but the relative growth rate increased to 0.6 at the 10^−^^2^ dilution. Further dilutions (10^−3^ to 10^−7^) stimulated fungal growth instead of inhibiting it (Figure 1). Amistar 250 SC with azoxystrobin (250 g/L) inhibited *F. graminearum* growth at concentrations ranging from 10^−^^1^ to 10^−^^3^ dilutions, with a consistent relative growth rate of 0.2. At higher dilutions (10^−4^ to 10^−^^6^), partial growth inhibition was observed, with relative growth rates increasing from 0.53 to 0.83. The 10^−7^ dilution had no measurable effect on fungal growth (Figure 1). Micosar 60 SL containing metconazole (CAS:125116-23-6) (60 g/L) strongly inhibited growth in the 10^−1^ dilution. Almost complete inhibition was observed at dilutions from 10^−^^2^ to 10^−4^, with relative growth rates ranging from 0.00 to 0.04. Higher dilutions (10^−^^5^ to 10^−^^7^) did not significantly affect fungal growth (Figure 1). Fungimat with tebuconazole (25 g/L) exhibited the strongest fungicide activity among the fungicides tested. Growth was nearly completely inhibited at dilutions from 10^−^^1^ to 10^−^^5^. At a 10^−^^6^ dilution, growth was moderately inhibited, with a relative growth rate of 0.5. The 10^−^^7^ dilution, however, had no effect on fungal growth (Figure 1).

### 2.2. Antifungal Interaction of CS and Selected Fungicides

**Figure 2:**
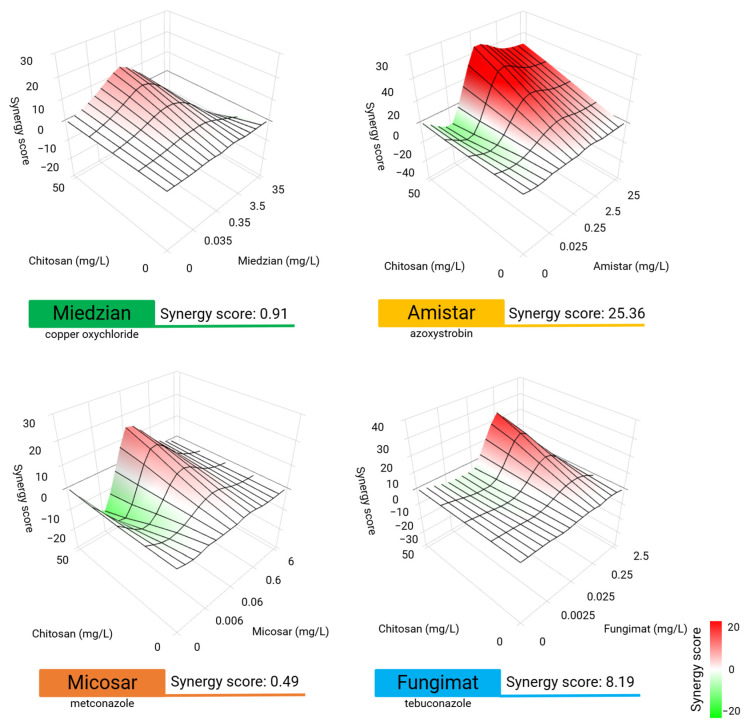
Surface graphs that show synergy scores for chitosan (CS) combined with four fungicides: Miedzian Extra 350 SC, Amistar 250 SC, Micosar 60 SL, and Fungimat. The concentrations of active ingredients in each fungicide are indicated, with CS used at a fixed concentration of 50 mg/L. Synergy scores for each combination were calculated using the web-based SynergyFinder 3.0 application [19].

CS, a biocompatible macromolecule with potent antifungal properties, was selected based on previous findings demonstrating its strong antifungal activity [1]. The study aimed to evaluate the effect of CS on *F. graminearum* growth in PDB medium supplemented with CS and one of the following fungicides: Miedzian Extra 350 SC, Amistar 250 SC, Micosar 60 SL, or Fungimat. The combination of Miedzian Extra 350 SC (0, 0.035, 0.35, 3.5, and 35 mg/L) and CS (0 and 50 mg/L) yielded an average synergy score of 0.91. Synergistic interactions (highlighted in red on the 3D graph) were observed at Miedzian concentrations between 0.35 and 3.5 mg/L when combined with CS at 50 mg/L. Additive effects (marked in white) were observed at Miedzian concentrations of 0.035 and 35 mg/L with CS at 50 mg/L (Figure 2). The combination of Amistar 250 SC (0, 0.025, 0.25, 2.5, and 25 mg/L) and CS (0 and 50 mg/L) resulted in an average synergy score of 25.36. Strong synergistic effects (marked in red) were observed at Amistar concentrations of 2.5 and 25 mg/L with CS at 50 mg/L. However, antagonistic interactions (highlighted in green) occurred at lower Amistar concentrations of 0.025 and 0.25 mg/L with CS at 50 mg/L (Figure 2). The combination of Micosar 60 SL (0, 0.006, 0.06, 0.6, and 6 mg/L) and CS (0 and 50 mg/L) demonstrated a weak synergistic interaction at a concentration of Micosar of 0.6 mg/L with CS at 50 mg/L, resulting in a synergy score of 0.49. Antagonistic interactions (marked in green) were observed at lower Micosar concentrations of 0.006 and 0.06 mg/L with CS at 50 mg/L (Figure 2). The combination of Fungimat (0, 0.0025, 0.025, 0.25, and 2.5 mg/L) and CS (0 and 50 mg/L) produced an average synergy score of 8.19, with strong synergy observed at the Fungimat concentration of 0.25 mg/L with CS at 50 mg/L. Additive interactions (highlighted in white) were observed for all other concentrations (Figure 2).

**Figure 3:**
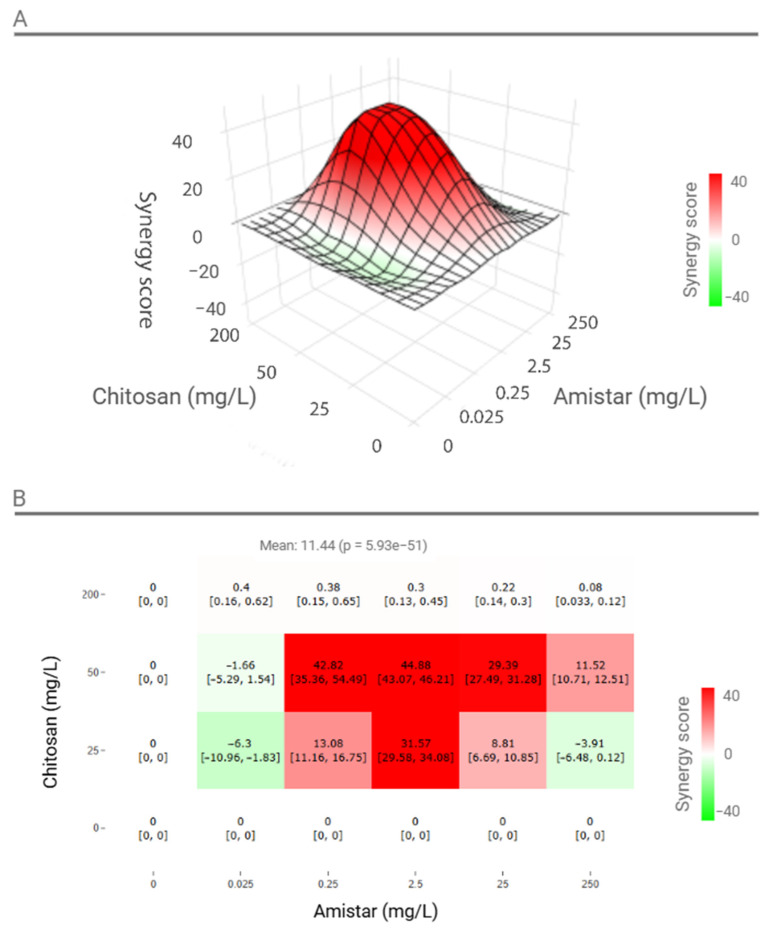
Surface graph showing the synergy scores for five concentrations of the active ingredient in Amistar 250 SC (0.025, 0.25, 2.5, 25, and 250 mg/L) combined with three concentrations of chitosan (CS; 25, 50, and 200 mg/L) to inhibit the growth of *Fusarium graminearum* in liquid PDB medium (**A**). The corresponding interaction matrix (**B**) is provided to illustrate the distribution of synergistic (red), antagonistic (green), and additive (white) interactions across the tested concentrations. The graph highlights the highest synergy scores and visually represents the dynamics of the interaction between CS and Amistar.

Based on the synergy scores determined for the four fungicides, further investigations were conducted that focused on Amistar 250 due to its pronounced synergistic potential. These studies evaluated five concentrations of Amistar (0.025, 0.25, 2.5, 25, and 250 mg/L) combined with three concentrations of CS (25, 50, and 200 mg/L) (Figure 3). The average global synergy score for these specified concentrations was 11.44. CS at 50 mg/L exhibited strong synergy with Amistar at concentrations of 0.25, 2.5, 25, and 250 mg/L, with the highest synergy scores observed at 0.25 mg/L (42.82) and 2.5 mg/L (44.88). CS at 25 mg/L also showed synergistic interactions with Amistar at 0.25, 2.5, and 25 mg/L but exhibited antagonistic effects at 0.025 mg/L and 250 mg/L (Figure 3B).

### 2.3. Genes Encoding ATP Binding Cassette (ABC) Proteins Identified in the F. graminearum Genome

**Figure 4:**
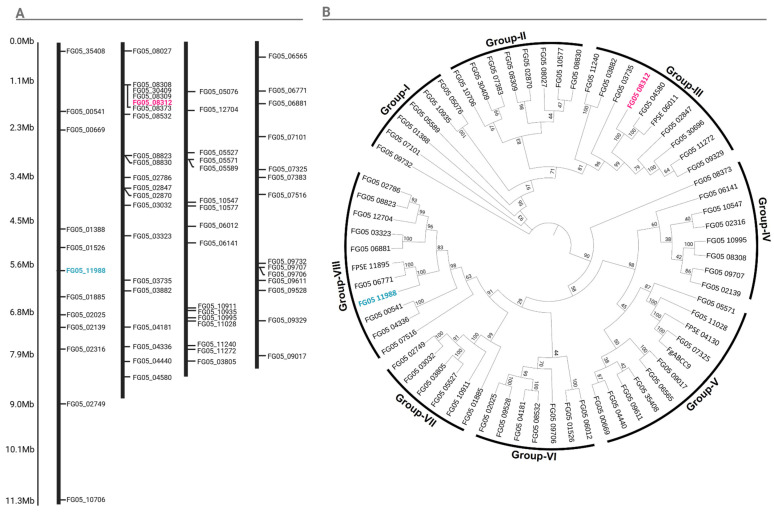
Genes encoding ABC transporter proteins in the *Fusarium graminearum* genome. (**A**) Chromosomal locations of the ABC transporter genes. (**B**) Phylogenetic analysis of ABC transporter proteins in the *Fusarium graminearum* genome using the maximum likelihood method. To distinguish them, previously identified genes are marked in their respective colors (FG05_11988 in cyan and FG05_08312 in pink).

A BLAST search was performed against the *F. graminearum* genome to identify previously unreported ABC proteins. The completeness of these proteins was verified and their target domains were confirmed using the NCBI Conserved Domain Database (NCBI CDD) (https://www.ncbi.nlm.nih.gov/Structure/cdd/wrpsb.cgi, accessed on 7 October 2024). This analysis identified a total of 63 ABC proteins (Table 1). The genes encoding these ABC proteins, designated *FgABC1* to *FgABC63*, were named according to their chromosomal locations (Figure 4A). For example, the gene FG05_00541, located at the start of chromosome 1, was named *FgABC1*, where “1” indicates the first ABC protein-encoding gene, “*Fg*” denotes *F. graminearum*, and “ABC” refers to the conserved domain characteristic of the ABC transporter subfamily. This naming convention was consistently applied to the remaining 62 ABC proteins. ABC protein-encoding genes were identified on all four chromosomes of the *F. graminearum* genome. Chromosome 2 contained the highest number of these genes (20 *FgABC* genes), followed by chromosome 3 (16 *FgABC* genes) and chromosome 4 (14 *FgABC* genes). Chromosome 1 harbored the fewest ABC protein-encoding genes, with 12 FgABC genes (Figure 4A).

### 2.4. Phylogenetic Analysis of ABC Transporter Proteins

Previous studies have identified four genes encoding ABC proteins, including FPSE_06011 (ZEB2-regulated ABC transporter 1), ZAR1 [17], and FPSE_11895 (ABC multidrug transporter, MDR1) [17], as playing roles in fungicide response. The newly identified ABC proteins were classified into eight groups based on their structural features (intron-exon distribution) and physicochemical properties (protein size and amino acid number) (Figure 4B). For example, genes encoding ABC proteins with a high number of introns and exons were classified into groups III, IV, and VIII, with no significant variation in the number of amino acids observed among these groups. On the contrary, smaller ABC proteins, characterized by fewer introns and exons and shorter amino acid sequences, were grouped into groups VI and VII. Group I showed an unusual distribution due to significant variation in gene structure and size. Groups II and V, though containing proteins with a relatively larger amino acid count, had fewer introns and exons within each group, suggesting a distinct gene structure compared to the other categories.

Group III includes the previously reported FPSE_06011/FG05_04580 (ZEB2-regulated ABC transporter1, ZAR1) and its homolog, FG05_08312, identified in the *F. graminearum* isolate under investigation. DNASTAR and phylogenetic analysis revealed that FG05_08312 is closely related to FPSE_06011/FG05_04580, leading to the selection of FG05_08312 as the primary ABC transporter gene for further studies (Supplementary Figure S1). Group VIII includes the previously reported FPSE_11895/FG05_06771 (ABC multidrug transporter, MDR1) and FG05_11988 from the *F. graminearum* isolate that is being studied in our lab. Based on sequence similarity, FPSE_11895 (ABC multidrug transporter, MDR1) and FG05_06771 are considered the same gene in different strains. Therefore, we selected FG05_11988, the closest homolog to FPSE_11895 (ABC multidrug transporter, MDR1), for further studies (Supplementary Figure S2). The genes FG05_08312 and FG05_11988 were selected as targets for dsRNA studies as potential genes associated with tolerance to fungicides.

### 2.5. Treatment with Amistar and CS Modifies the Expression of Selected Genes

**Figure 5:**
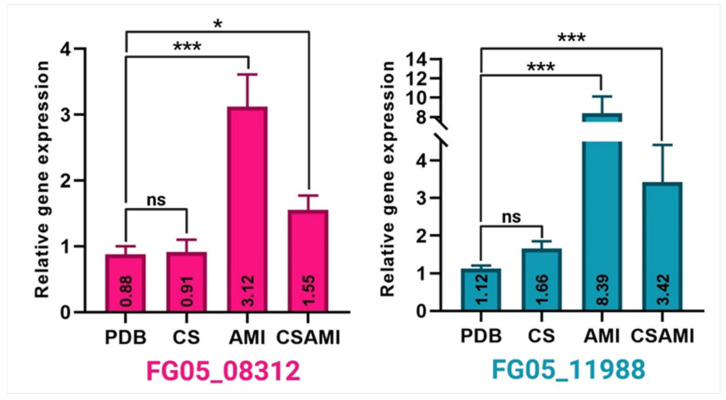
Relative expression of two *Fusarium graminearum* genes FG05_08312 and FG05_11988 in response to treatment with Amistar (AMI) and chitosan (CS) treatment 5 days post-inoculation (dpi). Significance markers are as follows: *** *p* ≤ 0.001, * *p* ≤ 0.05.

To analyze changes in the expression of potential target genes (FG05_08312 and FG05_11988) related to *F. graminearum’s* response to Amistar (azoxystrobin), relative gene expression was assessed across different treatments. For the FG05_08312 gene, the baseline expression in the PDB control treatment was approximately 0.88. Treatment with CS maintained a gene expression relative to that of the PDB control, while treatment with Amistar (AMI) resulted in a significant increase in expression to 3.12, the highest level observed for this gene across all treatments. Notably, co-treatment with CS and AMI (CSAMI) led to a significant downregulation in expression to 1.55 compared to AMI treatment alone (Figure 5). The expression pattern of the FG05_11988 gene was similar. The baseline expression level in PDB treatment was 1.12. Treatment with CS slightly but not significantly increased expression to 1.66. Treatment with AMI significantly upregulated the gene to 8.39. Consistent with the trend observed for FG05_08312, co-treatment with CSAMI resulted in an upregulation of FG05_11988 to 7.02, which, while still higher than in the PDB and CS treatments, was significantly lower than the expression level seen with AMI treatment alone (Figure 5).

### 2.6. Fusarium Transcriptome Response to Co-Treatment with CS and AMI

**Figure 6:**
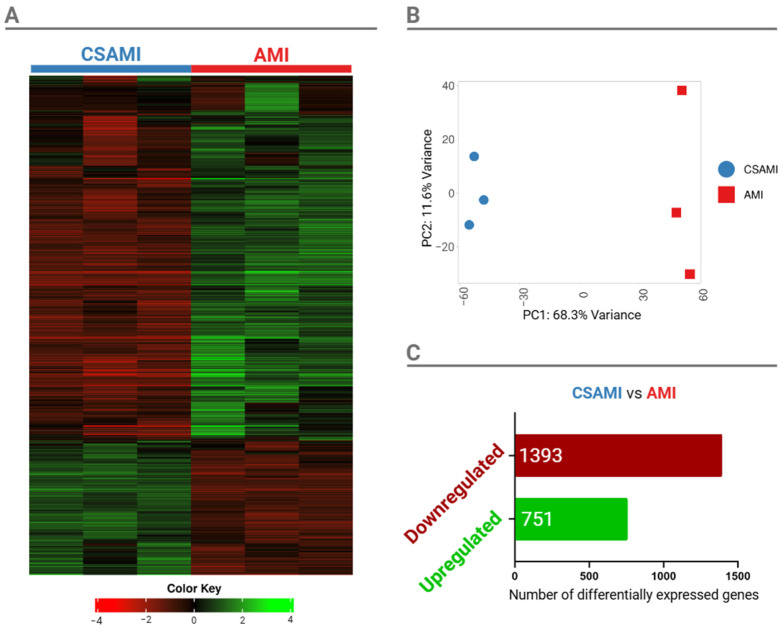
Hierarchical clustering heatmap of *Fusarium graminearum* cultures treated with chitosan and Amistar (CSAMI) versus Amistar alone (AMI). Each column represents an individual biological replicate (**A**). Upregulated genes are indicated in green, while downregulated genes are shown in red. Principal component analysis (PCA) of three biological replicates per treatment (**B**). Number of differentially expressed genes (DEGs) in *Fusarium graminearum* treated with chitosan and Amistar relative to those treated with Amistar alone (**C**).

The transcriptome of *F. graminearum* was analyzed under two conditions: a culture treated with AMI alone and a culture co-treated with AMI and CS. The culture served as a reference for observing the transcriptomic changes. Initial transcriptomic changes were examined using hierarchical clustering, based on Pearson’s average distance between the top 2000 genes (Figure 6A). Results of principal component analysis (PCA) indicated that the first principal component (PC1) accounted for 68.3% of the variance, effectively distinguishing samples according to their type of treatment (Figure 6B). Genes with a false discovery rate (FDR) < 0.05 and a log2 fold change > 2 were considered differentially expressed genes (DEG). Co-treatment with CS and AMI resulted in the downregulation of 1393 genes and the upregulation of 751 genes compared to treatment with AMI alone (Figure 6C).

Differentially expressed genes (DEGs) were further analyzed using gene set enrichment analysis (GSEA) to identify enriched pathways across the tested samples with an FDR cutoff value of <0.05. Pathways were ranked by normalized enrichment score (NES), where positive scores indicate upregulation and negative scores indicate downregulation.

**Figure 7:**
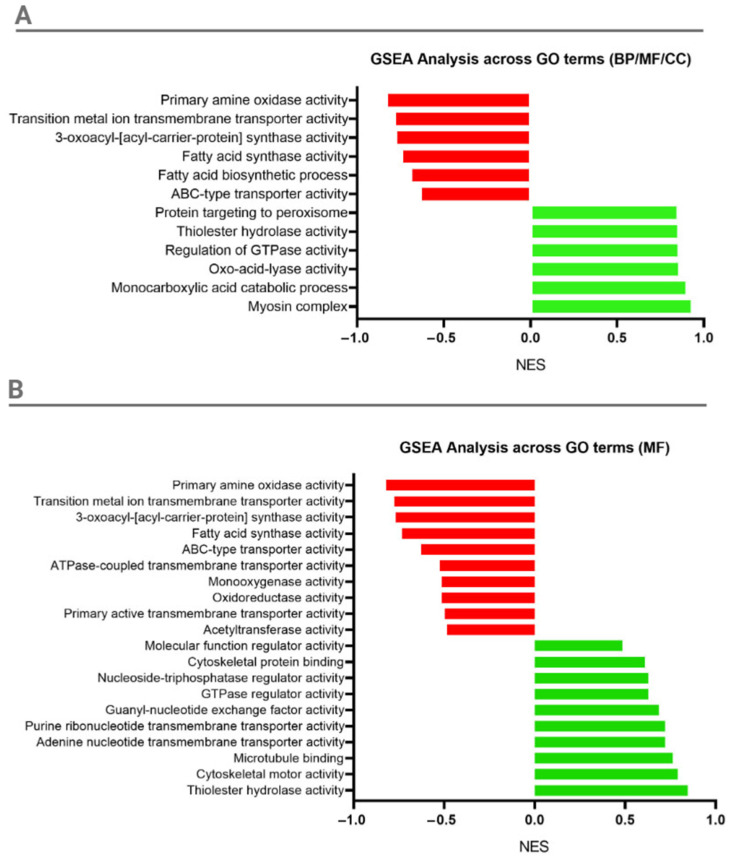
GSEA analysis of enriched Gene Ontology (GO) terms between CSAMI and AMI variants. (**A**) Enrichment of the top six upregulated and downregulated pathways across combined GO categories (Biological Process, Molecular Function, Cellular Component). (**B**) Top ten upregulated and downregulated GO terms within the Molecular Function category. Positive normalized enrichment score (NES) values indicate upregulation (marked in green color), while negative NES values indicate downregulation (marked in red color) of the specified pathways. Only pathways with an FDR < 0.05 were included in the analysis.

The top six pathways with the highest positive NES values were as follows: “Myosin complex”, “Monocarboxylic acid catabolic process”, “Oxo-acid-lyase activity”, “Regulation of GTPase activity”, “Thiolester hydrolase activity”, and “Protein targeting to peroxisome”. These pathways indicate increased activity in cytoskeletal function and specific metabolic processes related to acid catabolism (Figure 7A). The top six pathways with the most negative NES values were as follows: “Primary amine oxidase activity”, “Transition metal ion transmembrane transporter activity”, “3-oxoacyl-[acyl-carrier-protein] synthase activity”, “Fatty acid synthase activity”, “Fatty acid biosynthetic process”, and “ABC-type transporter activity”, indicating a decrease in the activity of processes such as amine oxidation, ion transport, and fatty acid biosynthesis (Figure 7A).

Focusing exclusively on Molecular Function (MF) GO terms with FDR < 0.05, the top 10 positively and top 10 negatively scored NES pathways were selected. The “Thiolester hydrolase activity” showed the highest positive NES, followed by pathways associated with cytoskeletal organization, motor function, and nucleotide transmembrane transporter activity. In the pathways with the most negative NES values, “Primary amine oxidase activity” exhibited the strongest downregulation, followed by “Transition metal ion transmembrane transporter activity”, “3-oxoacyl-[acyl-carrier-protein] synthase activity”, and “ABC-type transporter activity”, indicating that these pathways were the most downregulated across all GO terms. Additionally, pathways such as “ATPase-coupled transmembrane transporter activity”, “Primary active transmembrane transporter activity”, ”Oxidoreductase activity”, and “Monooxygenase activity” also exhibited negative scores, suggesting a general downregulation in genes associated with oxidative processes and transmembrane transport.

**Figure 8:**
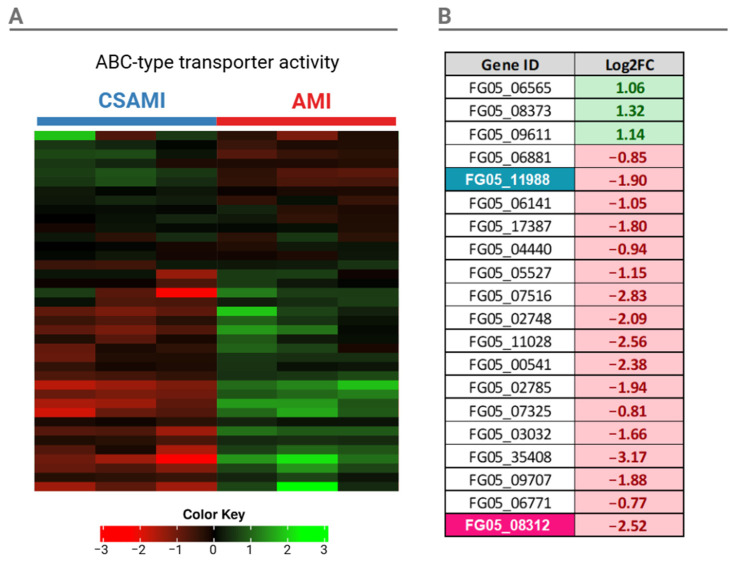
Hierarchical cluster heatmap of genes related to the GO term “ABC-transporter activity” under CSAMI and AMI treatment. Upregulated genes are indicated in green, while downregulated genes are shown in red (**A**). A table listing genes with FDR < 0.05, along with their respective log2 fold change (Log2FC) in relation to AMI treatment. Previously identified genes are marked in their respective colors (FG05_11988 in cyan and FG05_08312 in pink). Log2FC values greater than 0 are marked in green, while values less than 0 are marked in red (**B**).

Considering ABC (ATP-binding cassette) protein-coding genes, we conducted further analysis on the pathway “ABC-type transporter activity” due to its significant downregulation and potential link to the synergistic interaction between CS and AMI. Of the 39 genes annotated to this pathway, 20 exhibited significant changes in expression (FDR < 0.05) following co-treatment with CS and AMI (CSAMI) compared to treatment with AMI. Among these, 3 genes were upregulated, while 17 genes were downregulated. Upregulated genes associated with this pathway were FG05_06565, FG05_08373, and FG05_09611 (Figure 8).

### 2.7. dsRNA-Based Silencing of F. graminearum ABC Transporter-Encoding Genes

dsRNA-based silencing of *F. graminearum* ABC transporter-encoding genes results in significant gene silencing and strong retardation of *F. graminearum* growth. The use of dsRNA for RNA interference (RNAi)-based gene silencing is a powerful tool for functional genomics. In this study, we used this approach to silence *F. graminearum* genes that were strongly upregulated in response to AMI treatment. The ABC transporter-encoding genes FG05_08312 (group III) and FG05_11988 (group VIII) were selected for analysis, as they represent key genes in these groups. The most effective dsRNA for silencing each targeted gene was designed using the siRNA-Finder (si-Fi; version v1.2.3-0008) software [20]. The dsRNA designed for silencing FG05_08312 (group III) was a 393 bp fragment, spanning the 686 bp to 1079 bp region of the transcript (Supplementary Figure S2). The dsRNA for silencing FG05_11988 (group VIII) was a 413 bp fragment, spanning the 497 bp to 910 bp region of the transcript. Analysis indicated that these fragments would likely produce the most efficient siRNA molecules (Supplementary Figure S2).

**Figure 9:**
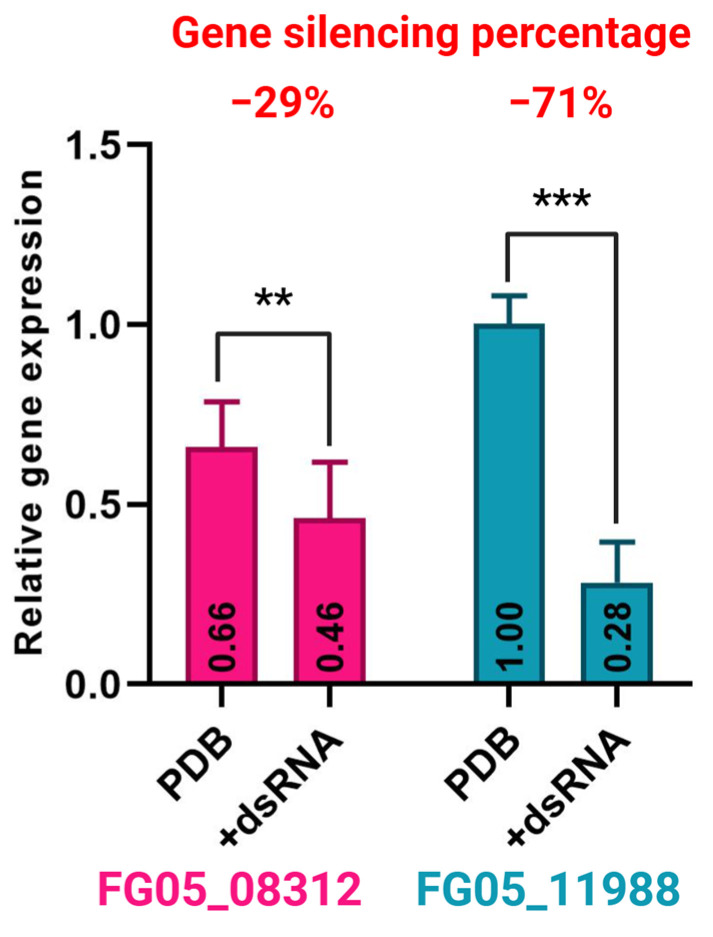
Relative expression of *Fusarium graminearum* genes FG05_08312 and FG05_11988 without dsRNA treatment (PDB) and with indicated dsRNA treatment (+dsRNA). Significance markers for *p*-value: *** *p* ≤ 0.001, ** *p* ≤ 0.01.

The designed and synthesized dsRNA was tested for gene silencing efficiency. For the dsRNA targeting the FG05_08312 gene (dsRNA FG05_08312), exogenous silencing resulted in a 29% reduction in relative expression compared to the nontreated variant. Similarly, dsRNA targeting FG05_11988 (dsRNA FG05_11988) led to a 71% decrease in relative expression of FG05_11988 (Figure 9).

### 2.8. Fungal Growth After Exogenous Gene Silencing, AMI, and CS

To investigate the interaction between the specified dsRNA and CS, AMI, and their combination (CSAMI), we used four variants of dsRNA treatment: no dsRNA (control), dsRNA targeting GFP (green fluorescent protein gene, acting as a control, i.e., targeting a gene not existing in *F. graminearum*), and dsRNA targeting two previously identified genes (FG05_08312 and FG05_11988). In the absence of dsRNA, the PDB treatment exhibited the highest OD_450_ value (1.00), serving as a relative baseline. CS treatment caused a moderate reduction in OD_450_ (0.87), while AMI led to a greater reduction (0.62). The combination treatment, CSAMI, showed the lowest value of OD_450_ (0.50), indicating the strongest effect in reducing fungal growth. All comparisons between treatment groups were statistically significant compared to the PDB control. When GFP-targeting dsRNA was introduced (serving as a control), the PDB group maintained a high OD_450_ value (1.06), similar to the condition without dsRNA. CS treatment caused a slight reduction (0.92), while AMI and CSAMI further reduced OD_450_ to 0.67 and 0.52, respectively. The statistical significance followed the same trends as in the no-dsRNA condition, with CSAMI being the most effective treatment.

**Figure 10:**
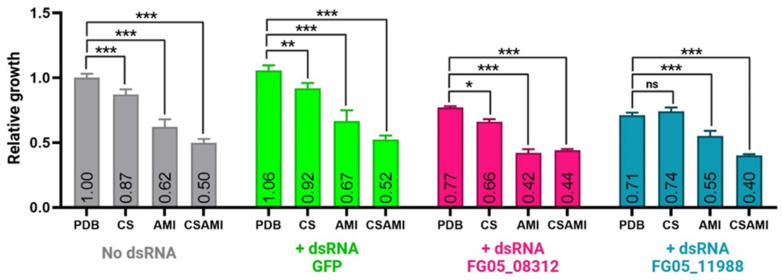
Relative growth values of *Fusarium graminearum* culture in PDB medium (PDB), PDB medium with 25 mg/L chitosan (CS), PDB medium with a final concentration of 0.25 mg/L of Amistar (AMI), and PDB medium with final concentrations of chitosan (25 mg/L) and Amistar (0.25 mg/L) (CSAMI). Significance markers for *p*-values: *** *p* ≤ 0.001, ** *p* ≤ 0.01, * *p* ≤ 0.05, and ns indicates no significant change.

When dsRNA targeting FG05_08312 was applied, OD_450_ values decreased across all treatments compared to the GFP control. The PDB group showed a moderate reduction to 0.77, while CS and AMI resulted in further decreases to 0.66 and 0.42, respectively. Combination treatment, CSAMI, achieved the lowest OD_450_ value at 0.44. Similarly, with dsRNA targeting FG05_11988, the OD_450_ values showed trends comparable to those observed for FG05_08312. The PDB group showed a reduction to 0.71, and CS remained at 0.74, with no significant differences (ns) between the two. In contrast, AMI reduced OD_450_ further to 0.55, while CSAMI again produced the lowest OD_450_ value at 0.40 (Figure 10).

## 3. Discussion

Fungicides vary in their effectiveness against *F. graminearum*, largely due to differences in their mechanisms of action and target specificity. For example, fungicides that inhibit ergosterol biosynthesis, such as Fungimat (tebuconazole) and Micosar (metconazole), disrupt the integrity of the fungal cell membrane. In contrast, azoxystrobin, present in AMI, targets mitochondrial electron transport, thereby impairing ATP production and leading to fungal cell death [21]. Experimental data on *F. graminearum* growth in PDB medium supplemented with these fungicides confirm their high efficacy. In contrast, copper oxychloride, an active component of Miedzian, functions as a multisite fungicide. It targets sulfhydryl (-SH) groups, causing enzyme inactivation and disrupting cell membranes. However, its antifungal effectiveness is generally lower compared to AMI, Micosar, and Fungimat. Data from *F. graminearum* growth assays show significant fungicidal activity only at the 10^−1^ and 10^−2^ dilutions, while lower concentrations were neutral or even stimulatory.

Combining fungicides with CS can result in various types of interaction, depending on the concentrations and specific fungicide used. When Miedzian (copper oxychloride) was applied at 33.5 mg/L in combination with 50 mg/L CS, the resulting synergy score was relatively weak, 0.91, indicating minimal improvement in antifungal activity. In contrast, other concentration combinations exhibited an additive effect, where the antifungal activity of both components combined without significant interaction. Previous studies have reported synergistic effects between copper-based fungicides and CS. For example, Lemke et al., 2022 demonstrated that the combination of copper fungicides with CS allowed for a 50% reduction in the required dose of the fungicide while maintaining effective plant protection [22]. This synergy suggests that CS may enhance the efficacy of copper-based fungicides, reducing the need for higher fungicide concentrations while still achieving desired levels of disease control.

The application of Micosar (metconazole) with CS resulted in two types of interactions: a weak synergistic effect (synergy score of 0.49) and an antagonistic effect at 0.006 mg/L Micosar and 50 mg/L CS. In contrast, the combination of Fungimat (tebuconazole) with CS primarily showed an additive effect, with a modest synergy score of 8.19 at 0.6 mg/L of Fungimat and 50 mg/L CS. When AMI (azoxystrobin) was applied with CS, the synergy was particularly strong, with a synergy score of 25.36 at concentrations ranging from 0.25 to 2.5 mg/L AMI and 50 mg/L CS. It was the highest score observed among all tested fungicides. Further testing with a broader range of concentrations revealed regions of strong synergy (synergy scores from 29.39 to 44.88), along with some antagonistic (−3.91 to −6.3 scores) and additive interactions. These findings align with the results of [23], who reported a high synergistic activity between CS and azoxystrobin. In their study, Amistar (azoxystrobin) and Signum (boscalid and pyraclostrobin) demonstrated the most significant increases in antifungal activity when combined with CS oligomers. In contrast, Teldor (fenhexamid) and Switch (cyprodinil and fludioxonil) showed a weaker response. When CS oligomers were used separately, they inhibited *Botrytis cinerea* germination by 4.8% at a concentration of 10 mg/L. However, in combination with Amistar (10 mg/L), the inhibition increased dramatically from 1.6% to 96.4%, and with Signum (2 mg/L), it increased from 1.7% to 89.0% [23]. The synergistic interaction between strobilurin-based fungicides, such as azoxystrobin or pyraclostrobin, and CS is not yet fully understood. Additionally, the precise antifungal mechanism of CS remains unclear, primarily due to the wide range of CS samples with varying properties, which results in diverse antifungal effects on different target organisms (review by [2]). Although the exact mode of action of CS is still under investigation, several hypotheses have been proposed. One of the most widely supported mechanisms is that the positively charged amine groups on CS interact with the negatively charged fungal cell membranes, leading to membrane destabilization [2].

In general, synergy between two compounds arises when their mechanisms of action are distinct and nonoverlapping. This may partially explain why synergy is observed with demethylation inhibitor (DMI) fungicides, although the interaction is not as pronounced as with strobilurin-based fungicides such as azoxystrobin and CS. Fungicides such as Fungimat (tebuconazole) and Micosar (metconazole) destabilize fungal cell membranes, a mechanism that overlaps with CS’s action, which could account for the weaker synergy observed. In contrast, strobilurin fungicides such as azoxystrobin disrupt cellular respiration, a mechanism that is not shared by CS. This distinct mode of action may explain the more pronounced increase in antifungal activity when azoxystrobin-based fungicides are combined with CS.

ABC transporter proteins play essential roles in various physiological functions, including cellular detoxification, signal transduction, and lipid regulation [14]. Emerging evidence highlights the significant roles of ABC transporters in *Fusarium* life cycle [24,25], pathogenesis [12,24,26,27] and responses to fungicides [17,18,24]. To further explore these roles, we identified the complete family of ABC-encoding genes in the *F. graminearum* genome and assigned names based on their chromosomal locations. Our analysis revealed a total of 64 genes distributed on the four *F. graminearum* chromosomes. Phylogenetic analysis grouped these genes into eight distinct clades. To the best of our knowledge, this study provides the first comprehensive characterization of the entire ABC transporter-encoding gene family in *F. graminearum* reported in the literature. The two ABC transporter-encoding genes, FG05_08312 and FG05_11988, were selected as targets for exogenous dsRNA-based silencing experiments due to their roles in fungicide resistance. Exogenous application of dsRNA to *F. graminearum* cultures effectively silenced the expression of these target genes (Figure 9) and inhibited fungal growth. When gene silencing was combined with simultaneous treatment with AMI and CS, a significantly stronger growth inhibition of *F. graminearum* was observed compared to treatment with CS or AMI alone. This finding suggests that the ABC transporters encoded by FG05_08312 and FG05_11988 genes, which are implicated in *F. graminearum* responses to CS and AMI, may also contribute to an as-yet unknown mechanism of *F. graminearum* resistance to azoxystrobin. Although the efficiency of gene silencing by specific dsRNAs must be considered, these results highlight the synergistic effect of dsRNA treatment and application of CSAMI (combined CS and AMI). Notably, gene-specific dsRNAs targeting FG05_08312 and FG05_11988 enhanced the fungicidal efficacy of the CSAMI treatment, underscoring their potential for improving fungal control strategies.

## 4. Materials and Methods

### 4.1. Materials

This study utilized various synthetic fungicides with different active ingredients. Miedzian Extra 350 SC, with copper oxychloride (CAS: 1332-65-6, active ingredient concentration: 350 g/L) as the active ingredient, was supplied by Synthos Agro (Oświęcim, Poland). Amistar 250 SC, containing azoxystrobin (CAS: 131860-33-8, active ingredient concentration: 250 g/L), was obtained from Syngenta Polska (Warsaw, Poland). Micosar 60 SL, with metconazole (CAS: 125116-23-6, active ingredient concentration: 60 g/L), was provided by Ciech Sarzyn S.A. (Nowa Sarzyna, Poland). Fungimat, which has tebuconazole (CAS: 107534-96-3, active ingredient concentration: 25 g/L) as its active ingredient, was supplied by SMB Life Science (Warsaw, Poland). CS, derived from shrimp with a viscosity of 10 cps, a molecular weight of 30 kDa, and a degree of deacetylation ≥ 90%, was supplied by Pol-Aura (Warsaw, Poland). PDB was supplied by ROTH (Karlsruhe, Germany). For dsRNA synthesis, the MEGAscript RNAi Kit was provided by Thermo Fisher Scientific (Waltham, MA, USA).

### 4.2. Methods

#### 4.2.1. CS Solution Preparation

A stock solution of CS was prepared by dissolving 1.5 g of specified CS in 500 mL of Milli-Q water with 1% acetic acid (pH 3.0) and stirring the mixture overnight at room temperature. The stock solution was diluted to the desired final concentration with Milli-Q water, and the pH was adjusted to 5.6 using NaOH. The solution was then filtered through a 0.22 μm filter.

#### 4.2.2. Production of *F. graminearum* Macroconidia

Macroconidia of the *F. graminearum* BW5 isolate were produced as described by [1]. Briefly, 3–4 cm^2^ of fresh *F. graminearum* mycelium obtained on PDA medium was used to inoculate 150 mL of an adapted V8 liquid medium containing 170 mL/L of potato and vegetable juice (Fortuna^®^, Pultusk, Poland) and 1.5 g/L calcium carbonate. The culture grew in 500 mL Erlenmeyer flasks on a shaker at 120 rpm at 25 °C under UV light for two weeks. The fungal suspension was filtered through Miracloth, and macroconidia were verified under a light microscope (Nikon^®^, Tokyo, Japan) and counted using a Fuchs–Rosenthal counting chamber. The macroconidia were suspended in ddH_2_O and stored at −80 °C.

#### 4.2.3. Microtiter Plate Fungal Growth Assay

To evaluate the impact of antifungal agents (fungicides, CS, dsRNA) on fungal growth, a 96-well microtiter plate assay was performed. Each well containing PDB and the tested antifungal agent was inoculated with 10 µL of *F. graminearum* isolate BW5 macroconidia suspension (1 × 10^5^ spores/mL). Plates were incubated in the dark at 25 °C for 5 days, and the optical density at 450 nm (OD_450_) of the culture was measured using a Tecan Infinite 200 Pro (Tecan, Männedorf, Switzerland). Wells containing all components except the *F. graminearum* inoculum served as background controls. The OD_450_ values were calculated using Equation (1):

OD_450_ = Measured OD_450_ − Control OD_450_ (1)

The minimum inhibitory concentration (MIC) of fungicides and CS was calculated to evaluate their respective antifungal efficacy. The MIC was defined as the lowest concentration that completely inhibited the growth of the *F. graminearum* BW5 isolate, as shown in Supplementary Table S2.

To compare the antifungal activity of across all treatments, OD_450_ values of the specified antifungal agents are presented as “relative growth” values, with the OD_450_ of the untreated *F. graminearum* culture serving as the reference point.

#### 4.2.4. RNA Extraction from Fungal Liquid Culture

The *F. graminearum* culture was incubated for 5 days in 5 mL of PDB medium supplemented with specified antifungal agents, with the same conditions as in the microtiter plates growth assay. The mycelium was rinsed with ddH_2_O, and total RNA was extracted from 100 mg of N_2_-frozen, homogenized mycelium using the Direct-zol RNA Miniprep Kit (Zymo-R2072, Irvine, CA, USA) according to the manufacturer’s protocol. RNA quantity was measured using a Nanodrop spectrophotometer (Nanodrop Technologies^®^, Wilmington, DE, USA). To confirm that the RNA was free of genomic DNA contamination, a 36-cycle PCR reaction was performed using 50 ng of DNase-treated RNA and primers targeting the *Fusarium Elongation factor1* gene (Supplementary Table S1). The absence of a PCR product after gel electrophoresis indicated that the RNA was free of gDNA. RNA quality was assessed using 1% agarose gel electrophoresis and verified with a Bioanalyzer 2100 (Agilent Technologies^®^, Santa Clara, CA, USA). The same method was used for both RT-qPCR and RNA-seq; a minimum of 2 µg of total RNA (RIN ≥ 6.5, 28S/18S ≥ 1.0, not contaminated with DNA, protein, or salt ions) was sent to the company to prepare an Illumina standard RNA library with polyA selection. The company HaploX Biotechnology Co., Ltd. (HaploX Biotechnology Co., Ltd., Hong Kong, China) performed Illumina NovaSeq 6000 sequencing with an estimated data output of ~25 M pair-end reads with a quality score of at least Q30. The raw FASTQ data obtained from sequencing was then further analyzed.

#### 4.2.5. RNA-Seq Results Quality Control and Analysis

The raw FASTQ data were quality-checked using FastQC version 0.12.1 (http://www.bioinformatics.babraham.ac.uk/projects/fastqc, accessed on 9 October 2024) with standard command-line parameters. Low-quality reads and adapter sequences were removed using Trimmomatic version 0.30 (http://www.usadellab.org/cms/?page=trimmomatic, accessed on 9 October 2024) [28]. Processed reads were aligned to the genome annotation file (GFF3) of the *F. graminearum* genome assembly (GCA_000240135.3), obtained from EnsemblFungi (https://fungi.ensembl.org/index.html, accessed on 9 October 2024), using HISAT2 version 2.2.1 with default settings [29]. The obtained output alignment file (.bam) was used as input for featureCounts to quantify transcript reads and further analyzed for differential expression using DESeq2 [30]. The cutoff values for differentially expressed genes (DEGs) were set at a false discovery rate (FDR; adjusted *p*-value) < 0.05 and a log2 fold change > 2 between the two experimental conditions. The iDEP 2.01 server (https://bioinformatics.sdstate.edu/idep/, accessed on 10 October 2024) [31] was used for principal component analysis (PCA), hierarchical cluster heatmap generation, and gene set enrichment analysis (GSEA) of differentially expressed genes [31]. The RT-qPCR was used to verify the reliability of the RNA-seq results (Supplementary Figure S4).

#### 4.2.6. Identification of the ABC Transporter in *F. graminearum* Strain CS3005 Genome

To identify the ABC transporter genes, we conducted a BLAST (https://blast.ncbi.nlm.nih.gov/Blast.cgi, accessed on 7 October 2024) [32] search of the protein sequence of the previously reported ABC transporter against the *F. graminearum* strain CS3005 genome database (https://fungi.ensembl.org/index.html; accessed on 9 October 2024) [33]. The identified proteins were analyzed using various web tools, including NCBI Conserved Domain Search (https://www.ncbi.nlm.nih.gov/Structure/cdd/wrpsb.cgi accessed on 7 October 2024), InterProScan (http://www.ebi.ac.uk/interpro/ accessed on 9 October 2024) [34], and SMART (http://smart.embl-heidelberg.de/ accessed on 10 October 2024) [35], to determine family-specific domains and motifs.

#### 4.2.7. Chromosomal Location and Multiple Sequence Alignment of ABC Transporter

Data concerning the chromosomal location were acquired from the *F. graminearum* strain CS3005 genome database (https://fungi.ensembl.org/index.html; accessed on 9 October 2024). The physical position of each ABC transporter on its respective chromosome was drawn by MapDraw [36]. The nomenclature of the identified ABC transporter was conducted in accordance with its chromosomal order. The multiple sequence alignment of the ABC transporter was conducted using DNASTAR (DNASTAR, Inc., Madison, WI, USA). The protein sequences utilized for the multiple sequence alignment were sourced from the *F. graminearum* strain CS3005 genome database (https://fungi.ensembl.org/index.html; accessed on 9 October 2024).

#### 4.2.8. Phylogenetic Analysis of the ABC Transporter

Phylogenetic analysis was conducted utilizing the MEGA 7.0.26 software tool [37]. The ABC proteins were initially aligned using the ClustalW v2.0 online program (http://www.ebi.ac.uk/Tools/webservices/services/msa/clustalw2_soap; accessed on 10 October 2024) [38]. The neighbor-joining approach was employed for tree construction. The bootstrap method with 1000 repeats was employed to assess the reliability of the built tree.

#### 4.2.9. Quantitative Reverse Transcriptase PCR (qRT-PCR) of Selected *F. graminearum* Genes

Total RNA extracted from fungal cultures was used for cDNA synthesis with oligo-dT primers and the RevertAid First Strand cDNA Synthesis Kit (Thermo Fisher Scientific^®^, Waltham, MA, USA), following the manufacturer’s instructions. The qPCR reaction mix consisted of 2 μL of 5× HOT FIREPol EvaGreen qPCR Mix Plus (ROX) (Solis Biodyne^®^, Tartu, Estonia), 0.3 μL of forward primer (10 μM), 0.3 μL of reverse primer (10 μM), 15 ng of template cDNA, and water to a final volume of 10 μL. Relative gene expression was calculated using the 2^−∆∆Ct^ method as described by [39]. All primers used for qPCR are listed in Supplementary Table S1. The results presented reflect the average relative expression values from at least three biological replicates and four technical repeats for each sample.

#### 4.2.10. In Silico Analysis and Synthesis of dsRNA

The SiFi 2.1 software (siFi21_1.2.3-0008, https://sourceforge.net/projects/sifi21/, accessed on 30 September 2024) Ref. [20] was used to select a region of the *F. graminearum* target gene transcript for dsRNA synthesis and to predict potential off-target sites. The *F. graminearum* genome assembly (https://www.ebi.ac.uk/ena/browser/view/GCA_901446245.1, accessed on 30 September 2024) was used for this analysis. The selected dsRNA region (Supplementary Figure S3) was synthesized using the MEGAscript Kit (Thermo Fisher Scientific, Waltham, MA, USA) following the manufacturer’s instructions. The template for dsRNA synthesis was obtained by PCR using forward and reverse primers with T7 promoter sequences (TAATACGACTCACTATAGGG) at their 5′ ends (listed in Supplementary Table S1). The PCR template was cDNA derived from RNA extracted from liquid fungal cultures as earlier described.

#### 4.2.11. Synergy Score Calculation

The OD_450_ values, indicating fungal growth in the presence of various fungicide and CS combinations, were analyzed and visualized using SynergyFinder 3.0 software [19] (https://synergyfinder.fimm.fi/synergy/20240912095621795773/) accessed on 12 August 2024. The Zero Interaction Potency (ZIP) model was applied to calculate the synergy score between CS and the fungicides.

#### 4.2.12. Statistical Analysis

The data in the graphs represent mean values ± standard errors. Each treatment was performed with at least three biological replicates. Analysis of variance (ANOVA) and least significant difference (LSD) post hoc tests were conducted and visualized using GraphPad Prism version 8.0.2 software (GraphPad Software, Boston, MA, USA). Results were considered statistically significant at *p* < 0.05.

## 5. Conclusions

Among the four tested fungicides, AMI, which contains the active ingredient azoxystrobin, demonstrated the highest synergy when combined with CS (30 kDa, degree of deacetylation < 90). This cotreatment resulted in the most significant increase in antifungal efficiency.

The combined application of dsRNA targeting ABC transporter genes and CSAMI (CS + AMI) further enhanced antifungal activity. This highlights a dual synergy: between CS and AMI and the role of dsRNA in silencing genes involved in the tolerance of *F. graminearum* to CSAMI treatment.

Our findings emphasize that exogenously induced RNAi via dsRNA is not only an effective antifungal strategy but also a powerful tool to investigate the roles of specific genes in fungal physiology, including tolerance to fungicides and CS, as demonstrated in this study.

This study also sheds light on the remarkable synergy between azoxystrobin and CS, a mechanism that remains to be fully understood but holds significant promise as a high-efficiency, ecofriendly approach for protecting plants against *F. graminearum* and potentially other fungal pathogens.

## Figures and Tables

**Table 1:** Members of the ABC transporter superfamily in the *Fusarium graminearum* genome.

Name	ID	Location	aa (Amino Acid)
*FgABC1*	FG05_35408	1:171,493–172,261	208
*FgABC2*	FG05_00541	1:1,656,768–1,660,879	1348
*FgABC3*	FG05_00669	1:2,114,494–2,119,292	1548
*FgABC4*	FG05_01388	1:4,539,563–4,542,964	1076
*FgABC5*	FG05_01526	1:4,989,585–4,992,166	810
*FgABC6*	FG05_11988	1:5,537,327–5,541,400	1280
*FgABC7*	FG05_01885	1:6,165,681–6,168,080	799
*FgABC8*	FG05_02025	1:6,632,333–6,634,291	618
*FgABC9*	FG05_02139	1:6,947,278–6,951,730	1328
*FgABC10*	FG05_02316	1:7,443,673–7,449,012	1463
*FgABC11*	FG05_02749	1:8,784,793–8,788,903	1139
*FgABC12*	FG05_10706	1:11,252,694–11,257,477	1434
*FgABC13*	FG05_08027	2:203,679–206,515	847
*FgABC14*	FG05_08308	2:974,238–978,799	1449
*FgABC15*	FG05_30409	2:980,752–982,170	473
*FgABC16*	FG05_08309	2:982,272–985,289	1005
*FgABC17*	FG05_08312	2:996,616–1,001,414	1517
*FgABC18*	FG05_08373	2:1,182,832–1,187,667	1611
*FgABC19*	FG05_08532	2:1,734,076–1,737,437	1103
*FgABC20*	FG05_08823	2:2,794,134–2,798,077	1263
*FgABC21*	FG05_08830	2:2,815,927–2,820,243	1405
*FgABC22*	FG05_02786	2:3,305,345–3,309,711	1282
*FgABC23*	FG05_02847	2:3,499,042–3,503,954	1470
*FgABC24*	FG05_02870	2:3,549,227–3,554,118	1614
*FgABC25*	FG05_03032	2:3,977,395–3,979,815	806
*FgABC26*	FG05_03323	2:4,770,610–4,774,676	1298
*FgABC27*	FG05_03735	2:5,870,874–5,877,578	1816
*FgABC28*	FG05_03882	2:6,111,507–6,116,198	1475
*FgABC29*	FG05_04181	2:7,003,497–7,007,051	1055
*FgABC30*	FG05_04336	2:7,491,208–7,495,365	1317
*FgABC31*	FG05_04440	2:7,828,733–7,833,347	1490
*FgABC32*	FG05_04580	2:8,248,117–8,252,691	1489
*FgABC33*	FG05_05076	3:1,062,050–1,065,121	1007
*FgABC34*	FG05_12704	3:1,513,794–1,517,788	1296
*FgABC35*	FG05_05527	3:2,556,404–2,559,580	1014
*FgABC36*	FG05_05571	3:2,706,262–2,711,406	1553
*FgABC37*	FG05_05589	3:2,766,585–2,768,514	625
*FgABC38*	FG05_10547	3:3,785,383–3,789,043	1145
*FgABC39*	FG05_10577	3:3,892,165–3,896,492	1404
*FgABC40*	FG05_06012	3:4,391,572–4,393,859	709
*FgABC41*	FG05_06141	3:4,780,606–4,785,758	1432
*FgABC42*	FG05_10911	3:6,384,333–6,386,479	698
*FgABC43*	FG05_10935	3:6,456,775–6,460,877	1351
*FgABC44*	FG05_10995	3:6,640,167–6,645,338	1610
*FgABC45*	FG05_11028	3:6,733,481–6,737,679	1382
*FgABC46*	FG05_11240	3:7,323,544–7,328,079	1457
*FgABC47*	FG05_11272	3:7,423,264–7,427,807	1461
*FgABC48*	FG05_03805	3:7,697,730–7,700,403	842
*FgABC49*	FG05_06565	4:418,689–422,612	1307
*FgABC50*	FG05_06771	4:1,148,013–1,152,169	1347
*FgABC51*	FG05_06881	4:1,529,213–1,533,756	1293
*FgABC52*	FG05_07101	4:2,326,597–2,328,684	607
*FgABC53*	FG05_07325	4:3,117,178–3,121,710	1452
*FgABC54*	FG05_07383	4:3,346,695–3,351,457	1552
*FgABC55*	FG05_07516	4:3,725,971–3,730,137	1358
*FgABC56*	FG05_09732	4:5,482,419–5,484,359	646
*FgABC57*	FG05_09707	4:5,586,196–5,591,334	1467
*FgABC58*	FG05_09706	4:5,592,046–5,594,437	727
*FgABC59*	FG05_09611	4:5,866,657–5,871,463	1514
*FgABC60*	FG05_09528	4:6,157,607–6,159,955	750
*FgABC61*	FG05_09329	4:6,849,754–6,854,425	1474
*FgABC62*	FG05_09017	4:7,753,696–7,758,794	1677
*FgABC63*	FG05_30696	KK082496:6,731–11,107	1240

## Data Availability

The raw RNA-seq data analyzed in this study are openly available in NCBI at https://www.ncbi.nlm.nih.gov/sra/PRJNA1199923; accessed on 19 December 2024.

## Supplementary Materials

The following supporting information can be downloaded at https://www.mdpi.com/article/10.3390/ijms26010262/s1. References [40,41] are cited in the Supplementary Materials.
