# Supplementary material for: The Synergy of Chitosan and Azoxystrobin Against Fusarium graminearum Is Modulated by Selected ABC Transporters
Source: Int J Mol Sci. 2024 Dec 30;26(1):262. doi: 10.3390/ijms26010262 (PMC11719997; doi:10.3390/ijms26010262)
Supplement: Supplementary file 1 [file ijms-26-00262-s001.zip › ijms-3371689-supplementary.pdf]

Supplementary Table S1. List of primers used for RT-qPCR and *in vitro* dsRNA synthesis.

	Gene ID	Primer sequence		Additional information
RT-qPCR	FG05_08312	FR	GCCAAGTCTATTGTCGCCCT	ABC-encoding gene
		rev	TCGATGCGCTGTTGACTTCT	
	FG05_11988	FR	CTACCTGACGGACTCGCAT	ABC-encoding gene
		rev	TGTCCGGTTCTTCTTGACCG	
	FG05_00007	FR	GTGCGGTTTTGACCAGATTT	Cytochrome synthesis gene
		rev	TATCAAGAGAGCGCAGCAGA	
	FGSG_06605	FR	TCTGGAGTCATTGCTTGTGC	Starch synthesis pathway gene
		rev	GCGAAAGGCCAGTTGTAGAG	
	FG05_08811	FR	CCTCCAGGATGTCTACAAGA	Ubiquitin C-terminal hydrolase - **housekeeping** gene (Kim et al. 2021)
		rev	CTCAACGGACTTGACTTCAG	
	FG05_01231	FR	GTTCTCGAGGCCAGCAAAAAGTCA	Elongation factor 1 alfa - **housekeeping gene** (Harris et al.. 2016)
		rev	CGAATCGCCGTTAGGGGTGTCTG	
dsRNA synthesis	dsRNA FG05_08312	FR	TAATACGACTCACTATAGGGCCAAGGCTTGAACAGGAA	dsRNA synthesis template for targeting FG05_08312 gene, T7 promoter sequence underlined
		rev	TAATACGACTCACTATAGGGAATTCGATGGCGTTGGCA	
	dsRNA FG05_11988	FR	TAATACGACTCACTATAGGGTCTTCAGGAAGCCATCAC	dsRNA synthesis template for targeting FG05_11988 gene, T7 promoter sequence underlined
		rev	TAATACGACTCACTATAGGGATGATCCTGTCCAGAACG	

Kim S, Park J, Kim D, Choi S, Moon H, Young Shin J, Kim JE, Son H. **Development of a versatile copper-responsive gene expression system in the plant-pathogenic fungus *Fusarium graminearum*.** Mol Plant Pathol. 2021 Nov;22(11):1427-1435. doi: 10.1111/mpp.13118. Epub 2021 Aug 13. PMID: 34390122; PMCID: PMC8518565.

Harris, L. J., Balcerzak, M., Johnston, A., Schneiderman, D., & Ouellet, T. (2016**). Host-preferential Fusarium graminearum gene expression during infection of wheat, barley, and maize.** Fungal Biol, 120(1), 111-123.

Supplementary Table S2. Minimum inhibitory concentration (MIC) of each active ingredient tested against *Fusarium graminearum in vitro* culture (PDB medium).

Active ingredient	MIC [mg/L]
copper oxychloride	35000
azoxystrobin	>25000
metconazole	6
tebuconazole	0.25
chitosan (CS_10)	100

Supplementary Table S3. Active ingredient content in each tested fungicide dilution.

		Active ingredient concentration in tested dilutions [mg/L]						
**Fungicide**	**Active ingredient**	**10^{-1}**	**10^{-2}**	**10^{-3}**	**10^{-4}**	**10^{-5}**	**10^{-6}**	**10^{-7}**
Miedzian	copper oxychloride	35000	3500	350	35	3.5	0.35	0.35
Amistar	azoxystrobin	25000	2500	250	25	2.5	0.25	0.25
Micosar	metconazole	6000	600	60	6	0.6	0.06	0.06
Fungimat	tebuconazole	2500	250	25	2.5	0.25	0.025	0.025

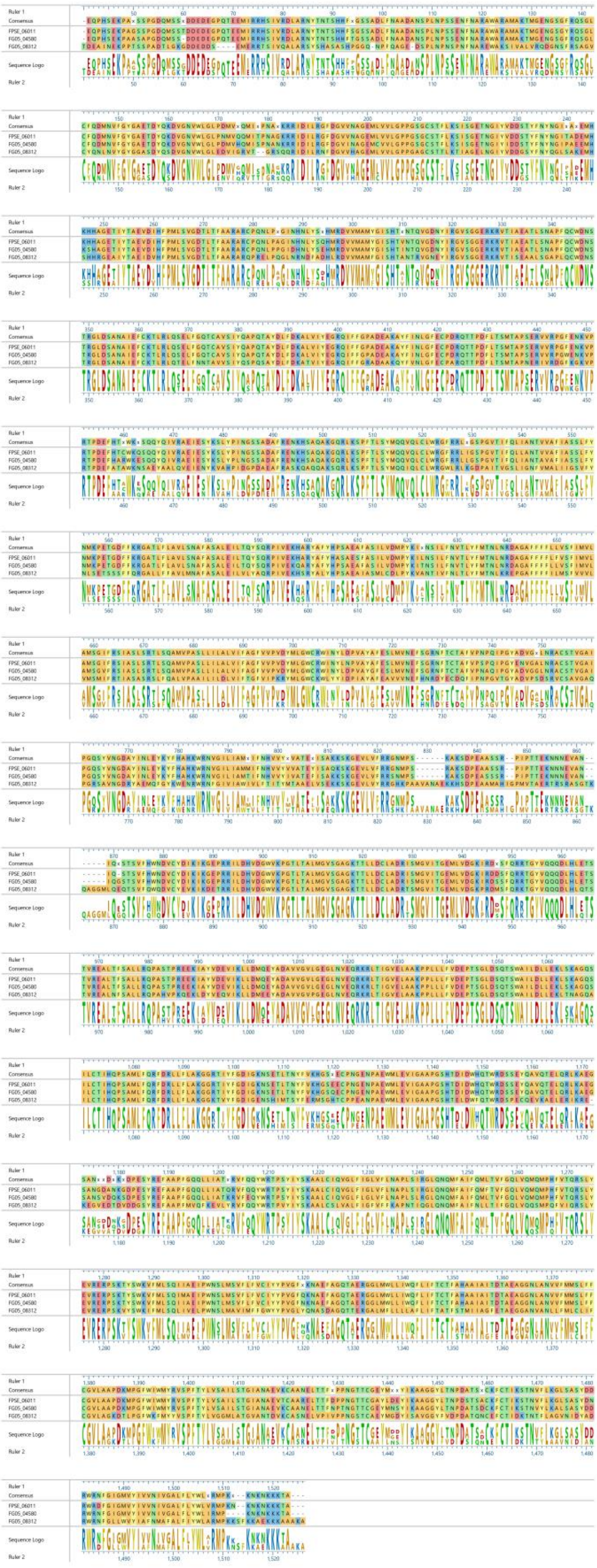

Figure S1. Multiple sequence alignment of FPSE_06011/FG05_04580 (ZEB2-regulated ABC transporter 1, **ZAR1**) and FG05_08312 sequences.

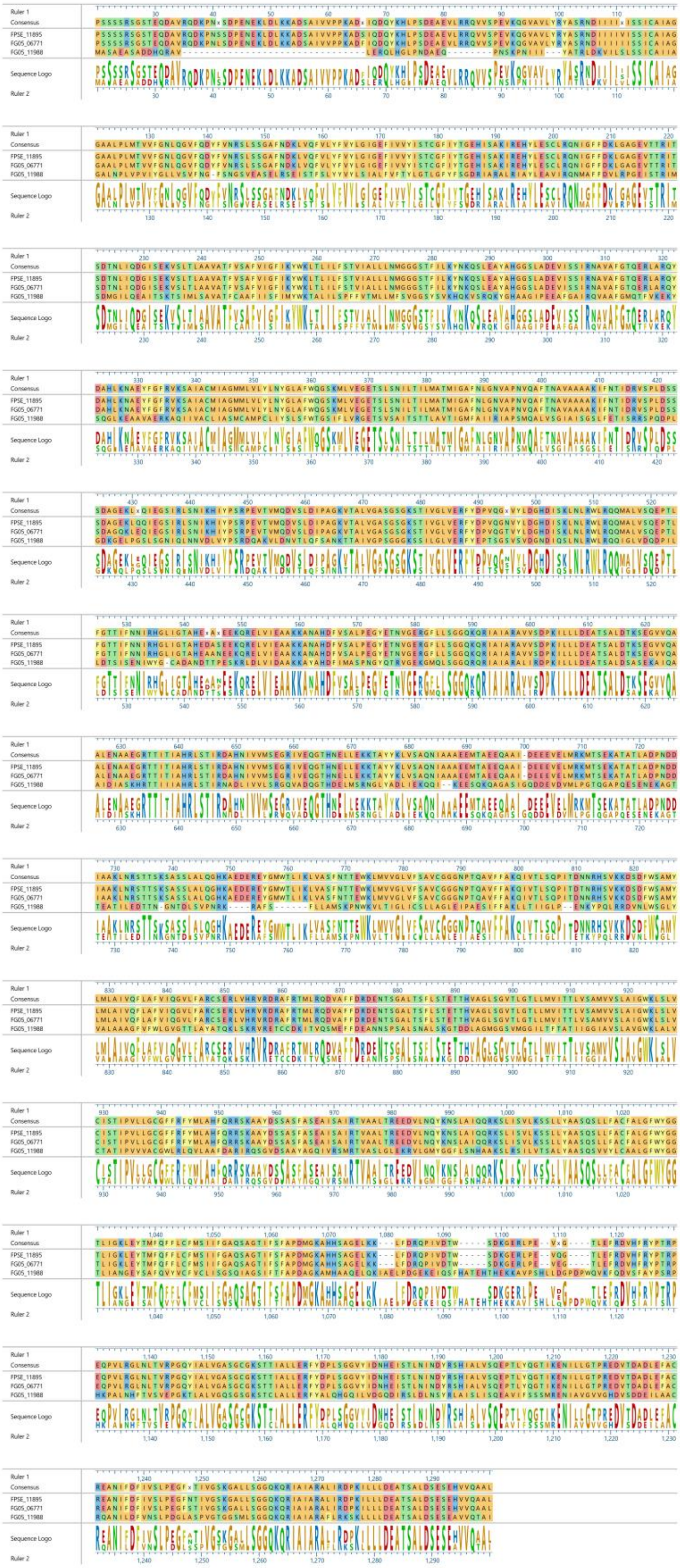

Figure S2. Multiple sequence alignment of *FPSE_11895* (ABC multidrug transporter, **MDR1**), *FG05_06771*, and *FG05_11988* sequences.

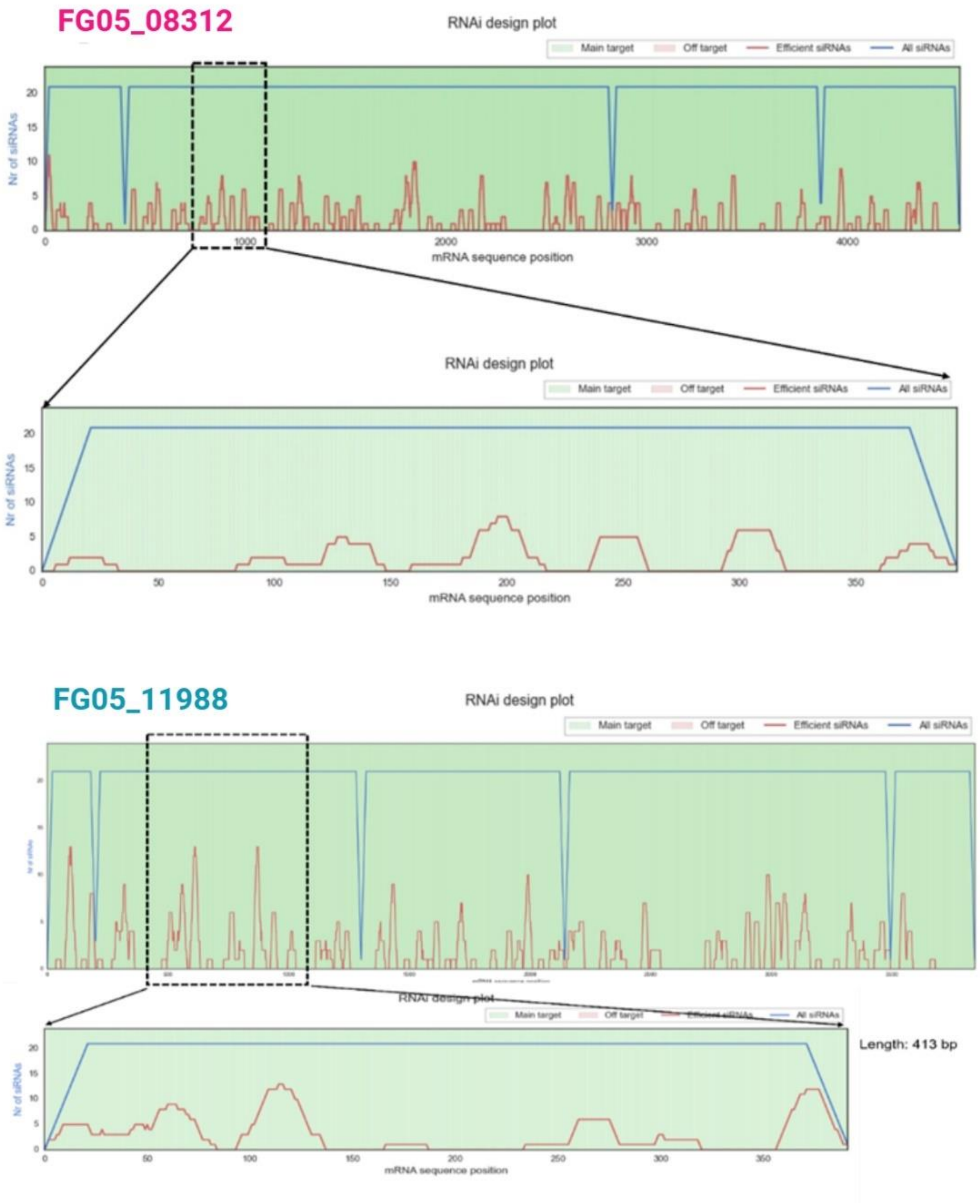

Figure S3. Distribution of siRNA efficiency along the mRNA nucleotide sequence of FG05_08312 (group III) and FG05_11988 (group VIII) genes.

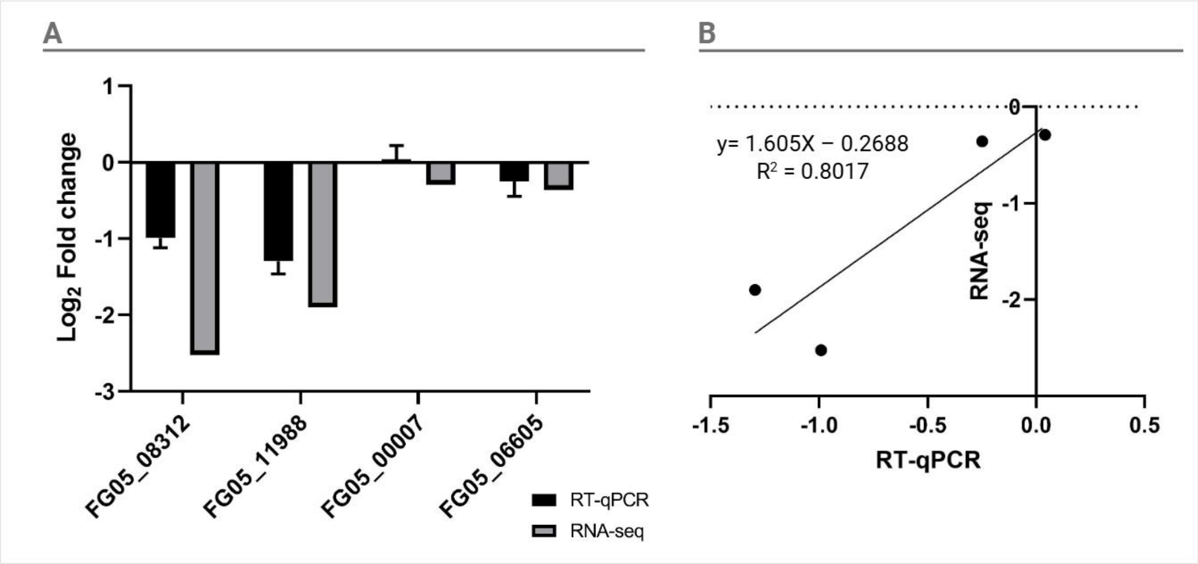

Figure S4. Validation of RNA-seq differentially expressed genes (DEGs) using RT-qPCR of four genes. The log2 fold change values (A) and the linear regression between the log2fold change of RNA-seq and RT-qPCR quantification are shown. Each data point corresponds to the results for a specific gene for RT-qPCR and RNA-seq (B).
